# Dynamics of circulating follicular helper T cell subsets and follicular regulatory T cells in rheumatoid arthritis patients according to HLA-DRB1 locus

**DOI:** 10.3389/fimmu.2022.1000982

**Published:** 2022-12-13

**Authors:** Paola V. Ferrero, Luisina I. Onofrio, Cristina del Valle Acosta, Estefania R. Zacca, Nicolas E. Ponce, Eduardo Mussano, Laura B. Onetti, Ignacio I. Cadile, Alicia B. Costantino, Marina L. Werner, Luciana A. Mas, Teresita Alvarellos, Carolina L. Montes, Eva V. Acosta Rodríguez, Adriana Gruppi

**Affiliations:** ^1^ Laboratorio de Inmunología, Hospital Nacional de Clínicas, Facultad de Ciencias Médicas, Universidad Nacional de Córdoba, Córdoba, Argentina; ^2^ Centro de Investigaciones en Bioquímica Clínica e Inmunología (CIBICI-CONICET), Facultad de Ciencias Químicas, Universidad Nacional de Córdoba, Córdoba, Argentina; ^3^ Servicio de Reumatología, Hospital Nacional de Clínicas, Facultad de Ciencias Médicas, Universidad Nacional de Córdoba, Córdoba, Argentina; ^4^ Laboratorio de Histocompatibilidad, Hospital Privado Universitario de Córdoba e Instituto Universitario de Ciencias Biomédicas, Córdoba, Argentina

**Keywords:** rheumatoid arthritis, Tfh cells, Tfr cells, HLA-DRB1, DMARDs, DAS28-ESR

## Abstract

B cells, follicular helper T (Tfh) cells and follicular regulatory T (Tfr) cells are part of a circuit that may play a role in the development or progression of rheumatoid arthritis (RA). With the aim of providing further insight into this topic, here we evaluated the frequency of different subsets of Tfh and Tfr in untreated and long-term treated RA patients from a cohort of Argentina, and their potential association with particular human leukocyte antigen (HLA) class-II variants and disease activity. We observed that the frequency of total Tfh cells as well as of particular Tfh subsets and Tfr cells were increased in seropositive untreated RA patients. Interestingly, when analyzing paired samples, the frequency of Tfh cells was reduced in synovial fluid compared to peripheral blood, while Tfr cells levels were similar in both biological fluids. After treatment, a decrease in the CCR7^lo^PD1^hi^ Tfh subset and an increase in the frequency of Tfr cells was observed in blood. In comparison to healthy donors, seropositive patients with moderate and high disease activity exhibited higher frequency of Tfh cells while seropositive patients with low disease activity presented higher Tfr cell frequency. Finally, we observed that HLA-DRB1*09 presence correlated with higher frequency of Tfh and Tfr cells, while HLA-DRB1*04 was associated with increased Tfr cell frequency. Together, our results increase our knowledge about the dynamics of Tfh and Tfr cell subsets in RA, showing that this is altered after treatment.

## Introduction

Rheumatoid arthritis (RA) is a chronic autoimmune inflammatory disease characterized by symmetric progressive joint destruction associated with pain and synovial proliferation (1, 2). An altered adaptive immunity that involves various cell types and immunomodulatory molecules has been linked to the pathogenesis of the disease (3). Moreover, genetic and environmental factors may be involved in triggering the disease (1, 2). Among the genetic factors, several human leukocyte antigen (HLA)-DRB1 have been shown to be associated with RA susceptibility while others are thought to be protective (4). T cells, B cells and antigen-presenting cells aberrantly activated, together with the orchestrated interaction of pro-inflammatory cytokines, play key roles in the pathophysiology of RA (5). B cells are involved in RA progression by producing autoantibodies but also exert regulatory functions (6). Autoantibodies, including rheumatoid factor (RF) and anti-citrullinated peptide antibody (ACPA), are a hallmark of the disease. Based on their presence, RA patients can be subdivided into seropositive and seronegative. ACPA in RA patients has been shown to be associated not only with distinct genetic and environmental risk factors but also with a more severe disease course in seropositive patients (7, 8).

Follicular helper T (Tfh) cells are specialized suppliers of help to B cells and are essential for the differentiation process that ultimately gives rise to antibody-secreting cells. Tfh cells are distinguished by a high expression of C-X-C chemokine receptor type 5 (CXCR5), inducible co-stimulator (ICOS), programmed death-1 (PD-1), and B-cell lymphoma 6 (Bcl-6), as well as by Interleukin (IL)-21 secretion, all features that are crucial for their function (9, 10). Although germinal centers (GCs) in secondary lymphoid organs are the main effector site of Tfh cells, human blood contains CXCR5^+^ CD4^+^ T cells that share phenotypic and functional properties with GC Tfh cells and are, therefore, called “circulating” Tfh cells (10–16). Remarkably, exaggerated or dysregulated Tfh cell responses are linked to autoimmunity (17, 18).

Conflicting data have been reported on Tfh dynamics in RA patients. Tfh cells were found at higher frequency in blood from RA patients than from healthy donors (HD) (19, 20), and this T cell subset was identified in synovial fluid (SF) of inflamed joints (21). Furthermore, different subsets of circulating Tfh cells, as well as of activated B cells, correlated positively with Disease Activity Score in 28 Joints (DAS28) in RA patients (20). Regarding effector Tfh mediators, high levels of IL-21 mRNA transcripts were detected in peripheral blood mononuclear cells (PBMCs) from RA patients, and high levels of serum IL-21 correlated positively with DAS28 and the levels of serum anti- cyclic citrullinated peptide (anti-CCP) antibodies (22). In contrast, we found no differences between RA patients and HD in the percentages of CXCR5^+^ CD4^+^ T cells or in the frequencies of CXCR3^+^CCR6^−^ (Tfh1), CXCR3^-^CCR6^-^ (Tfh2), and CXCR3^-^CCR6^+^ (Tfh17) cell subsets in a small cohort of 24 patients from Argentina (23). In that opportunity, we also found a lack of correlation between CXCR5^+^ CD4^+^ T cells, Tfh1, Tfh2, and Tfh17 with DAS28 or the erythrocyte sedimentation rate (ESR). Similarly, it was reported that the frequency of CXCR5^+^ cells within a CD4^+^ T cell gate was not different among RA patients and normal individuals (24), although circulating Tfh cells from patients with RA, particularly those seropositive, showed significantly increased expression of the inhibitory receptor CD200. Also, these authors reported that Tfh subsets in RA patients were not polarized toward Th1, Th2 or Th17 phenotypes. Finally, another study reported few PD-1^hi^CXCR5^+^ Tfh cells in synovial tissue samples and in SF from seropositive RA patients (25). Considering these controversies, probably related to different strategies of Tfh cell characterization together with differences in the genetic background of patient cohorts, the treatment regimens, and the time span of disease, more in-depth studies are necessary to elucidate the role of Tfh cells in the physiopathology of RA.

Follicular regulatory T (Tfr) cells, a subpopulation of regulatory T cells, are critical mediators of Tfh cell response and antibody production in GCs. Tfr cells are distinguished from other CD4^+^ T cell subsets predominantly by their Forkhead box P3 (FoxP3) and CXCR5 expression (26, 27). Changes in the frequency of Tfr cells are associated with aberrant autoantibody production (28). Circulating Tfr cells are increased in RA patients who achieve a stable remission of disease in comparison with patients with active RA and healthy controls (HC) (29).

B cells, Tfh cells and Tfr cells are part of a circuit that, if unbalanced, can contribute to or sustain the pathogenesis of RA. To provide more insights about this topic, we evaluated here the frequency of different subtypes of Tfh and Tfr cells in untreated and long-term treated RA patients from a cohort of Argentina and their potential association with HLA class II antigens and disease activity.

## Material and methods

### Patients and healthy donors

Ninety-nine patients over 18 years diagnosed with RA according to American College of Rheumatology (ACR) and the European League against Rheumatism (EULAR) 2010 criteria (30), untreated and under different treatment conditions, were recruited from the Rheumatology Service (Hospital Nacional de Clínicas, HNC). Forty-six sex- and age-matched HD were also recruited as controls. Exclusion criteria for RA patients and controls included known or suspected ongoing infection, neoplasia, other autoimmune or inflammatory diseases, vaccination within the last 2 months, pregnancy, corticotherapy >10 mg prednisone/day or equivalent. The characteristics of the RA patients and controls are summarized in **Table 1**. Treatments in the RA patients recruited included conventional synthetic disease-modifying antirheumatic drugs (csDMARDs), mainly methotrexate (MTX), Tumor Necrosis Factor (TNF) inhibitors (golimumab, adalimumab, etanercept, certolizumab) combined or not with MTX, and tofacitinib combined or not with MTX. The RA DAS28 using the erythrocyte sedimentation rate (DAS28-ESR) (31) was assessed at the time of peripheral blood (PB) collection. Twenty-seven patients diagnosed with RA, including patients with recent diagnosis and naïve of specific treatment as well as patients with established RA without specific treatment for at least six months, were followed up after 3 months from the start of therapy. Treatment response was evaluated according to EULAR response criteria (32). Patients with good or moderate response to treatment were classified as responders and patients with no response as non-responders. Patients were classified as low disease activity (DAS28-ESR ≤3,2) and moderate/high disease activity (DAS28-ESR >3,2) according to Aletaha et al. (33).

**Table 1:** Demographic and clinical characteristics of RA patients and healthy donors.

			RA					
					Treatment			
	HD(n=46)	All pts (n=99)		*P value*	Untreated(n= 52)	csDMARDs (n=24)	TNF Inh (n=14)	Tofa(n=9)
**Age,*^a^* years**	50 ± 13	54 ± 14		0.14*^c^*	52 ± 15	54 ± 13	53 ± 14	62 ± 10
**Sex, n° F/M**	39/7	84/15		0.81*^d^*	46/6	17/7	12/2	9/0
**WBC, *^a^* n°.10^9^/L**	6.2 ± 1.5	7.8 ± 2.7		**<0.0001** *^e^*	7.9 ± 2.4	8.3 ± 3.6	6.6 ± 2.2	6.2 ± 2.2
**Hb, *^a^* g/L**	13.6 ± 1.3	13.0 ± 1.9		**0.028** *^e^*	12.8 ± 1.5	13.2 ± 2.8	12.9 ± 1.8	13.6 ± 1.2
**Plat, *^a^* n°.10^9^/L**	226 ± 56	278 ± 91		**0.0001** *^e^*	279 ± 91	286 ± 96	264 ± 88	258 ± 95
**ESR,** *^b^* **mm/h**	7(4-11)	18(6-34)		**<0.0001** *^c^*	21(9-37)	9(4-24)	16(3-30)	13(7-25)
**CRP,** *^b^* **mg/L**	3(1-5)	6(3-20)		**<0.0001** *^e^*	7.5(3.0-24.2)	7.0(3.0-35.5)	4.1(2.0-9.4)	6.0(1.0-11.3)
**DAS28-ERS** *^a^*	–	4.12 ± 1.30		–	4.64 ± 1.12	3.87 ± 1.46	3.16 ± 0.91	3.39 ± 1.27
**RF, n° (+/-)**	1/45	67/32		**<0.0001** *^d^*	39/13	13/11	8/6	7/2
**Anti-CCP,** *^b^* **U/mL**	2.6(1.5-5.0)	71.0(5.2-1150.0)		**<0.0001** *^e^*	157.5(7.8-1400.0)	34.0(3.7-1519.0)	45.0(5.2-320.0)	55.0(3.1-207.5)

^a^expressed by mean ± SD; ^b^expressed by median and interquartile range (IQR); P value All pts vs HD; significant p values are in bold; ^c^Student´s t-test; ^d^Chi-square test; ^e^Welch´s test.

RA, Rheumatoid Arthritis; HD, Healthy Donors; pts, patients; csDMARDs, conventional synthetic Disease Modifying Anti-Rheumatic Drugs; TNF inh, Tumor Necrosis Factor inhibitors drugs; Tofa, tofacitinib; F/M, Female/Male; WBC, White Blood Cells; Hb, Hemoglobin; Plat, Platelets; ESR, Erythrocyte Sedimentation Rate; CRP, C-Reactive Protein; DAS28, Disease Activity Score in 28 joints; RF, Rheumatoid Factor (RV<1/20); anti-CCP, anti-cyclic citrullinated peptide (RV<20U/mL).

The study had the approval of the HNC Institutional Committee of Ethics of Health Research and the Council for Ethical Evaluation of Health Research of the Cordoba government and was conducted according to the Declaration of Helsinki on studies with human subjects. Written informed consent was obtained from patients and controls.

### Samples

PB samples anticoagulated by ethylenediamine tetraacetic acid (EDTA)-K2, by sodium citrate 3.8% W/V, or by heparin were collected for different studies. Serum samples were obtained for immunological laboratory tests. When required, PBMCs were isolated by Ficoll-Hypaque density gradient centrifugation (GE Healthcare Bio-Science AB, Uppsala, Sweden) and cryopreserved until used. Synovial fluid (SF) was collected in heparinized tubes from patients with active disease whenever possible.

### Erythrocyte sedimentation rate, C-reactive protein, rheumatoid factor and anti-cyclic citrullinated peptide antibody determination

EDTA-K2 and sodium citrate 3.8% W/V anticoagulated PB samples were collected, for cytological analysis and for erythrocyte sedimentation rate (ESR) determination, respectively. Serum C-Reactive Protein (CRP) concentration was determined by particle-enhanced turbidimetric immunoassay using the Dimension RxL Max SIEMENS autoanalyzer (New York, EE. UU.). RF was determined by a latex agglutination test (Wiener Laboratories SAIC, Santa Fe, Argentina) considering positive titer ≥1/20; and anti-CCP highly sensitive antibodies were quantified by Enzyme-Linked Immunosorbent Assay (Orgentec Diagnostika Gmbh, Mainz, Germany) and values ≥20 UI/mL were considered positive.

### Flow cytometry

For phenotypic studies, cells from PB or SF were stained with a mixture of fluorochrome-labeled monoclonal Abs, indicated in **Supplementary Table 1**, following the procedure previously described (34, 35). The gating strategies used to identify the different cell subsets evaluated during this study are depicted in **Figures S1** for PB and S2 for SFMCs.

For intracellular protein staining, including cytokines and transcription factors, cryopreserved PBMCs were thawed and maintained overnight in complete RPMI medium 1640 supplemented with 10 ng/mL of recombinant human IL-2 (BioLegend, San Diego, United States). For intracellular cytokine staining, rested PBMC were stimulated with 50ng/ml phorbol 12-myristate 13-acetate (Sigma-Aldrich, St. Louis, USA) plus 1ug/ml ionomycin (Sigma-Aldrich, St. Louis, USA) in the presence of Brefeldin A (BD GolgiPlug™ RUO, BD, United States), as we previously described (35). After stimulation, cells were washed and stained for the identification of surface molecules. Subsequently, cells were washed, fixed and permeabilized for 1 hour at 4°C using fixation/permeabilization solution (eBioscience™ FOXP3 staining buffer set, Invitrogen, Carlsbad, San Diego, United States) and stained for 30 min at room temperature with anti-IFN-γ, anti-IL-17A and anti-FOXP3 (**Supplementary Table 1** and gating strategy in **Figure S2C**). The intracellular expression of CTLA-4, granzyme B (GzmB), and FOXP3 in Tfr cells was determined in PBMCs following the same protocol for intracellular cytokine staining without stimulation (**Supplementary Table 1** and gating strategies in **Figure S3**). Finally, stained cell samples were acquired on a Becton Dickinson FACSCanto II Flow Cytometer (BD, United States) and analyzed with FlowJo software (version 10).

### HLA DRB1 typing

The HLA DRB1 typing was made in 95 RA patients and 43 HD. Deoxyribonucleic acid (DNA) was isolated from PB anticoagulated by EDTA-K2 in a QIACube robotic workstation using a QIAmp DNA Blood Mini Kit (QIAGEN, Hilden, GE). Luminex-based technology was applied to discriminate between the different HLA antigens as described in Citera et al. (36). The typing method consisted in a Sequence Specific Oligonucleotide (SSO) primed PCR assay (Labtype SSO Typing, One Lambda Inc., CA, USA). Specific software was used to assign positive or negative reactions based on the strength of the fluorescent signal. The assignment of the HLA typing was based on positive and negative probe reactions compared with published HLA gene sequences (4, 36).

### Statistics

Statistical analyses were performed with GraphPad Prism 7 software and MedCalc 10.2.0.0 software. *P* values <0.05 (two-sided) were considered significant. The Kolmogorov-Smirnov test was initially performed to determine the distribution of the datasets. The specific tests used are indicated in the legends for figures.

## Results

### Frequency of total Tfh cells and different Tfh cell subsets in RA patients

**Figure 1:**
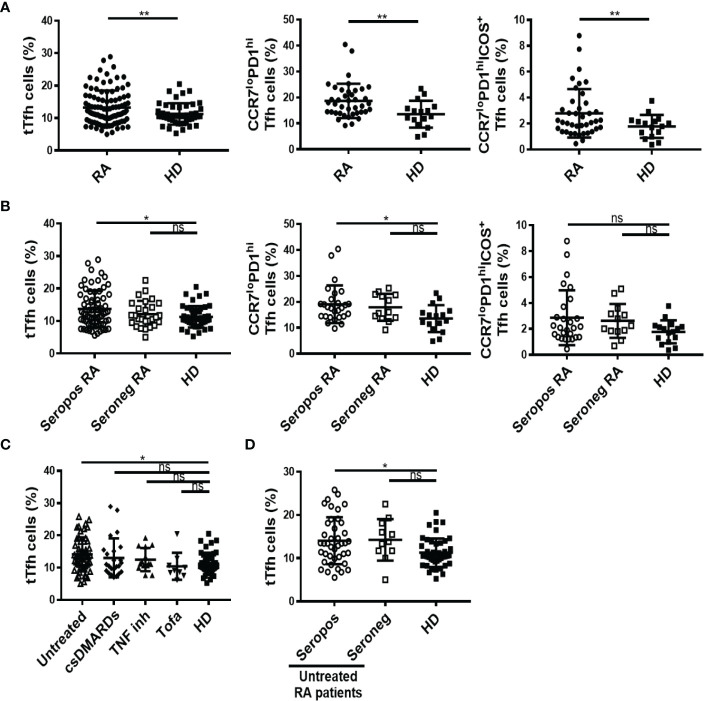
Frequency of total Tfh cells and different Tfh cell subsets in RA patients. **(A)** Frequency of CD25^lo^CD127^hi^ total (t) Tfh cells (left), CCR7^lo^PD1^hi^ Tfh cell subset (middle) and activated CCR7^lo^PD1^hi^ICOS^+^ Tfh cell subset (right) in RA patients (n=99, 40 and 40, respectively) and HD (n=46, 16 and 16, respectively). **(B)** Frequency of tTfh cells in seropositive (Seropos, n=72) and seronegative (Seroneg, n=27) RA patients and HD (n=46) (left), frequency of CCR7^lo^PD1^hi^ Tfh cell subset (middle) and activated CCR7^lo^PD1^hi^ICOS^+^ Tfh cell subset (right) in seropositive (Seropos, n=27) and seronegative (Seroneg, n=13) RA patients and HD (n=16). **(C)** Frequency of tTfh cells in untreated (n=52), patients treated with different drugs: csDMARDs (n=24), TNF inhibitors (TNF inh, n=14), tofacitinib (Tofa, n=9), and HD (n=46). **(D)** Frequency of tTfh cells in Seropos (n=41), Seroneg (n=11) untreated RA patients and HD (n=46). Symbols represent individual subjects. Mean ± DS is shown. *P* values were determined by Welch-test or T-Test in **(A)**. One-way Analysis of Variance (ANOVA) followed by Bonferroni Multiple Comparisons Test were used in **(B, D)** and Kruskal-Wallis followed by Dunn’s Multiple Comparisons Test were used in **(C)**. Two-tailed probability, *p < 0.05, **p < 0.01; ns, not significant.

As a first step to elucidate the features of Tfh cell responses during RA, we determined the frequency of circulating Tfh cells in a cohort of RA patients from Argentina. Circulating Tfh cells are used as accessible counterparts of lymphoid tissue Tfh cells in human studies (10, 12, 16, 37), due to restrictions in obtaining cells from human secondary lymphoid tissues. Of note, a subset of circulating CXCR5^+^CD4^+^ T cells characterized by the phenotype C-C chemokine receptor type 7 (CCR7)^lo^PD1^hi^ is reported to differentiate into mature Tfh cells upon antigen encounter and to promote GC responses in mice and humans, resembling active Tfh cells from secondary lymphoid organs (12). Using flow cytometry, we identified total Tfh (tTfh) as the population of CD25^lo^CD127^hi^ cells within the circulating CXCR5^+^ CD45RA^neg^ CD4^+^CD3^+^ T cells, following the gating strategy depicted in **Figure S1A**. In addition, we identified Tfh cell subsets reported to be efficient helpers (15) according to the phenotype CCR7^lo^PD1^hi^ and activated CCR7^lo^PD1^hi^ICOS^+^ Tfh cells, with the gating strategy described in **Figure S1B**. We determined that the frequency of tTfh cells, as well as the frequency of CCR7^lo^PD1^hi^ and CCR7^lo^PD1^hi^ICOS^+^ Tfh subsets were significantly higher in RA patients than in HD (**Figure 1A**). When the analysis was performed in RA patients classified by serological status, we observed that the frequencies of tTfh and CCR7^lo^PD1^hi^ Tfh cells were higher in seropositive patients (with anti-CCP and/or RF) than in HD, while the frequency of these populations in seronegative patients (without anti-CCP or RF) did not present significant differences with respect to HD (**Figure 1B**). On the other hand, the frequency of the CCR7^lo^PD1^hi^ICOS^+^ cell subset did not show differences when the RA patients were analyzed by serological status. No significant differences were observed in the absolute number of tTfh cells between RA patients and HD (**Figure S1C**). The absolute numbers of tTfh cells determined in our cohort of patients were similar to those reported by Chakera et al. (24).

To evaluate the impact of different treatments for RA on Tfh populations, we analyzed the frequency of Tfh cells in untreated and treated RA patients. Although with limitations derived from the small sample size in patients undergoing treatments with TNF inhibitors and tofacitinib, we found that the frequency of tTfh cells was significantly higher only in untreated patients when compared to HD (**Figure 1C**). Moreover, among the untreated patients and in comparison with HD, tTfh cells were more frequent in seropositive than in seronegative patients (**Figure 1D**). These differences were not observed in tTfh cells absolute numbers, which were similar in RA patients and HD, regardless of their serological status (**Figure S1D**) or the treatment received (**Figure S1E**). We also evaluated the frequency of tTfh cells at the inflammation site, analyzing SF (**Figure S2A**). By analyzing paired samples from eleven RA patients, we determined that the frequency of tTfh cells was lower in SF than in PB (**Figure S2B**).

We next evaluated the functional capability of Tfh cells to produce IL-17, an effector function associated with enhanced helper function (10, 15). Thus, in a small group of randomly selected patients, we evaluated IL-17- and interferon (IFN)γ-producing tTfh cells using the gating strategy indicated in **Figure S2C**. We detected a higher frequency of IL-17^+^IFNγ^neg^ Tfh cells in RA patients vs HD, while the frequency of the IL-17^neg^IFNγ^+^ cell subset was similar in both groups of subjects (**Figure S2D**).

These results analyzed together suggested that, within our cohort of RA patients, the frequency of tTfh cell and Tfh cell subsets with a phenotype of enhanced helper function is higher in seropositive patients, particularly in those untreated. Considering this, we tried to establish a link between Tfh cell frequencies and the levels of autoantibodies. No correlations were found between the levels of anti-CCP antibodies and the frequency of tTfh cells, CCR7^lo^PD1^hi^ or CCR7^lo^PD1^hi^ICOS^+^ Tfh cell subsets in the complete cohort (**Table 2**). Also, no correlations were observed either when the analysis was limited to anti-CCP positive patients or to anti-CCP positive untreated patients (**Table 2**). Similar findings were obtained when the correlations were calculated using RF titers (**Table S2**).

**Table 2:** Correlation between anti-Cyclic Citrullinated Peptide antibody levels and different T follicular cell populations.

	All RA patients		Anti-CCP positive RA patients		Anti-CCP positive untreated RA patients	
	r	*p* value	r	*p* value	r	*p* value
**tTfh cells (%)**	0.025*^a^*	0.80	-0.079*^a^*	0.54	-0.31*^b^*	0.069
**CCR7^lo^PD1^hi^ Tfh** **cell subset (%)**	0.22*^b^*	0.16	0.23*^b^*	0.87	0.37*^b^*	0.17
**CCR7^lo^PD1^hi^ICOS^+^Tfh** **cell subset (%)**	0.12*^b^*	0.47	-0.031*^b^*	0.89	0.05*^b^*	0.86
**Tfr cells (%)**	0.25*^a^*	**0,012**	0,081*^a^*	0,54	0,062*^b^*	0,72

^a^Spearman’s coefficient of rank correlation (rho); ^b^Pearson´s Correlation coefficient r. Significant p value is in bold.

RA, Rheumatoid Arthritis; anti-CCP, anti-cyclic citrullinated peptide (RV<20U/mL).

### Frequency of Tfr cells in RA patients

**Figure 2:**
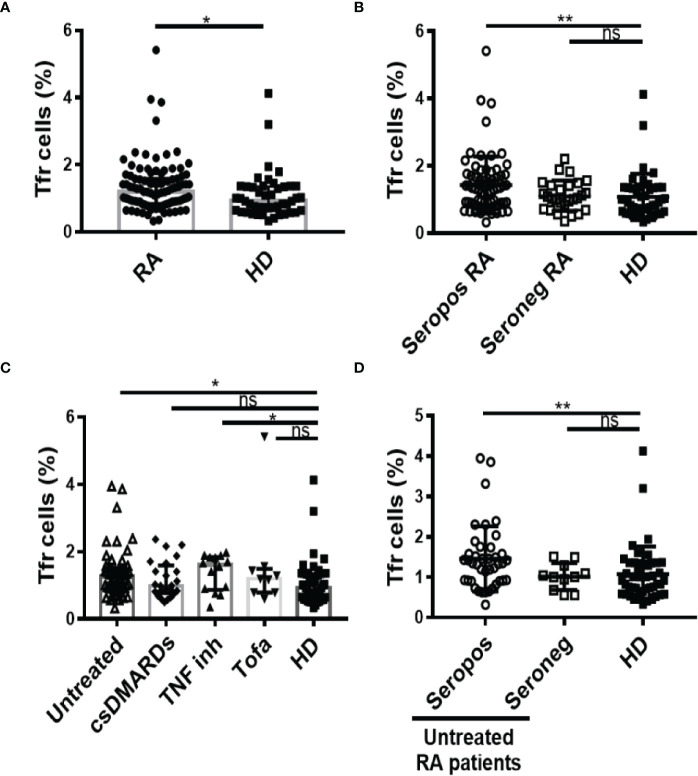
Frequency of Tfr cells in RA patients. **(A)** Frequency of Tfr cells in blood from RA patients (n=99) and HD (n=46). **(B)** Frequency of Tfr cells in seropositive (Seropos, n=72) and seronegative (Seroneg, n=27) RA patients and HD (n=46). **(C)** Frequency of Tfr cells in untreated (n=52), RA patients treated with different drugs: csDMARDs (n=24), TNF inhibitor (TNF inh, n=14), tofacitinib (Tofa, n=9) and HD (n=46). **(D)** Frequency of Tfr cells in Seropos (n=41) and Seroneg (n=11) untreated RA patients and HD (n=46). Symbols represent individual subjects. Mean ± DS is shown in **(B)** and **(D)**. Median ± interquartile range is shown in **(A)** and **(C)**; *P* values were determined by T-Test in **(A)**. Kruskal-Wallis Test followed by Dunn’s Multiple Comparisons Test were determined in **(B–D)**. Two-tailed probability *p < 0.05, **p < 0.01, ns, not significant.

The frequency of Tfr cells identified as CD25^hi^CD127^lo^ cells within the circulating CXCR5^+^CD45RA^neg^CD4^+^CD3^+^ T cell gate was determined through the strategy depicted in **Figure S1A**. We observed that RA patients from our cohort had a significantly higher frequency of Tfr cells than HD (**Figure 2A**). Phenotypic characterization of these Tfr cells from RA patients and HD showed marked similarities, which included expression of FoxP3 in around 90% of the subset, conserved expression of Cytotoxic T-Lymphocyte Antigen 4, and a low percentage of GzmB-expressing cells (**Figure S3**). When the RA patients were segregated according to the presence of autoantibodies, only seropositive patients exhibited a significantly higher frequency of Tfr cells in comparison to HD (**Figure 2B**). In relation to RA patients with different treatment regimens, we observed that the increased frequency of Tfr cells was particular to untreated and TNF inhibitor-treated patients (**Figure 2C**). Considering the small number of patients in the available sample, this observation needs to be confirmed with a greater number of patients. Of note, within the group of untreated RA patients, the significant increase in Tfr cells frequency was limited to seropositive patients (**Figure 2D**). These significant differences were partially extended to the Tfr cell absolute numbers, as we established that this parameter showed no significant differences when comparing HD with total RA patients or RA patients divided according to the treatment (**Figures S4A, S4B**) but was significantly increased in seropositive RA patients (**Figure S4C**). The absolute numbers of Tfr cells determined in our cohort of patients were similar to those reported by Fonseca et al. (28). Tfr cell frequency was weakly but positively correlated with serum anti-CCP antibody levels in the full cohort of RA patients but not when the analysis was limited the group of RA patients with anti-CCP, either all or untreated (**Table 2**). No correlation was found between the RF titer and the frequency of Tfr cells in the groups of patients evaluated (**Supplementary Table 2**). Interestingly, the frequency of Tfr cells in paired SF and PB from RA patients was similar (**Figure S4D**; gate strategy described in **Figure S2A**).

### Correlation between Tfh and Tfr cells in RA patients

**Figure 3:**
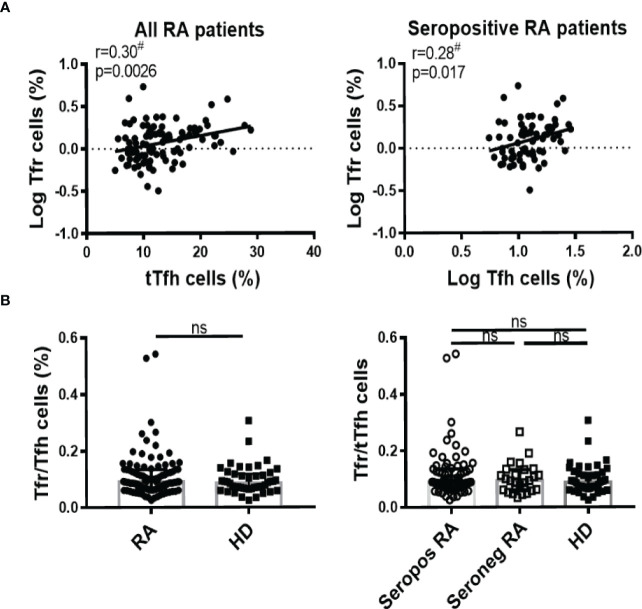
Correlation between Tfh and Tfr cells in RA patients. **(A)** Correlation between tTfh and Tfr cells frequency in all RA patients (n=99, left) or in seropositive RA patients (n=72, right). **(B)** Tfr/Tfh ratio in RA patients (n=99) vs HD (n=46) (left) or in seropositive (Seropos, n=72) and seronegative (Seroneg, n=27) RA patients and HD (right). Symbols represent individual subjects. ^#^Pearson’s correlation coefficient r and *P* values are shown in **(A)**; lines of best fit were drawn. Median ± interquartile range is shown in **(B)**; T-Test (left) and Kruskal-Wallis Test followed by Dunn’s Multiple Comparisons Test (right) were determined in **(B)**. ns, not significant.

Many works have implicated an imbalance in Tfr/Tfh ratio as responsible for the pathophysiology in RA patients (19, 38, 39). Considering this, we next aimed at establishing correlations between these subsets in the context of RA. We established that the frequency of Tfr cells showed a weak but statistically significant positive correlation with the frequency of tTfh cells in all patients analyzed together as well as in the group of seropositive patients (**Figure 3A**). There were no statistically significant differences in the Tfr/tTfh ratio between HD and RA patients, irrespective of whether they were seropositive or not (**Figure 3B**). As expected, the Tfr/tTfh ratio was significantly increased in SF compared to the PB of RA patients, because of decreased frequency of tTfh cells and unchanged frequency of Tfr cells in SF (**Figure S4D**).

### HLA-DRB1 antigens and its relationship with tTfh and Tfr cells in RA patients

**Figure 4:**
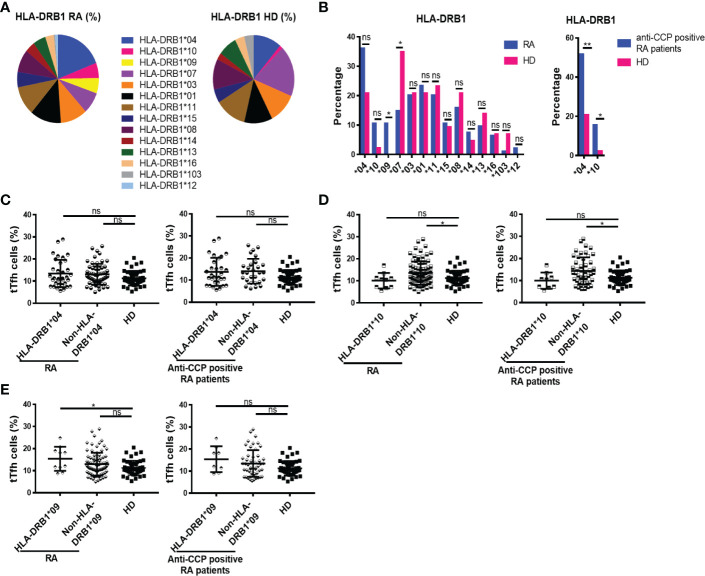
Distribution of HLA-DRB1 and its relationship with tTfh cells in RA patients. **(A)** HLA-DRB1 variants distribution in RA patients (n=94) and HD (n=43). **(B)** HLA-DRB1 variants frequency in all RA patients (n=94) and HD (n=43, left), and HLA-DRB1*04 or HLA-DRB1*10 frequency in RA patients with anti-CCP antibodies (n=58) and HD (n=43, right). **(C)** Frequency of tTfh cells in RA patients (n=94) and in RA patients with anti-CCP antibodies (n=58) divided by HLA-DRB1*04 presence (n=34 out of 94 RA patients and n=30 out of 58 patients with anti-CCP) and HD (n=43). **(D)** Frequency of tTfh cells in RA patients (n=94) and in RA patients with anti-CCP antibodies (n=58) divided by HLA-DRB1*10 presence (n=10 out of 94 RA patients and n=9 out of 58 patients with anti-CCP) and HD (n=43). **(E)** Frequency of tTfh cells in RA patients (n=94) and in RA patients with anti-CCP antibodies (n=58) divided by HLA-DRB1*09 presence (n=10 out of 94 RA patients and n=8 out of 58 patients with anti-CCP) and HD (n=43). Symbols represent individual subjects and mean ± DS is shown in **(C–E)**. Chi square test or Fisher Exact test were used in **(B)** as appropriate. *P* values were determined by Kruskal-Wallis test followed by Dunn’s Multiple Comparisons Test in **(C, D)**; One-way Analysis of Variance (ANOVA) and Bonferroni Multiple Comparisons Test were used in **(E)**. Two-tailed probability *p < 0.05, ns, not significant.

The risk of developing RA is known to be influenced by genetic factors, including certain HLA-DRB1 variants (4). HLA-DRB1, which encode the shared epitope that comprises a particular 5 amino acid domain, appear to confer susceptibility only for development of the subgroup of RA that is associated with ACPA (2). Based on this information, we wondered if the higher frequency of tTfh and Tfr cells detected in some RA patients could be associated with specific HLA variants. To address this, we first studied the profile of HLA-DRB1 antigens in our cohort of patients and then analyzed the frequency of tTfh and Tfr cells of the patients according to the different HLA-DRB1 locus. The pie charts in **Figure 4A** show the distribution of HLA-DRB1 antigens in the RA patient cohort and HD. In agreement with another study made in Argentina (36) and preview reports (4), the HLA-DRB1*09 was detected with a significantly higher frequency, while the HLA-DRB1*07 was underrepresented, in patients with diagnosis of RA in comparison to HD (**Figure 4B**). HLA-DRB1*04 and HLA-DRB1*10 showed a tendency to higher frequency in RA patients when the total cohort was analyzed, and their enrichment was statistically significant in patients with anti-CCP antibodies (**Figure 4B**). By analyzing the frequency of tTfh cells in RA patients by HLA-DRB1 presence, we observed that the frequency of tTfh cells was similar in HD and RA patients with or without HLA-DRB1*04, either in the total cohort or in the anti-CCP antibodies group (**Figure 4C**). In contrast, RA patients lacking HLA-DRB1*10 within the complete cohort, or in the group with anti-CCP antibodies, exhibited a significantly higher frequency of tTfh cells compared to HD (**Figure 4D**). The frequency of tTfh cells was also increased in RA patients with HLA-DRB1*09, another HLA variant overrepresented in our cohort of patients, compared to HD but not in patients without the HLA- DRB1*09. No differences were found in the frequency of tTfh cells in the group of patients with anti-CCP antibodies, regardless of whether patients had HLA- DRB1*09 or not (**Figure 4E**).

**Figure 5:**
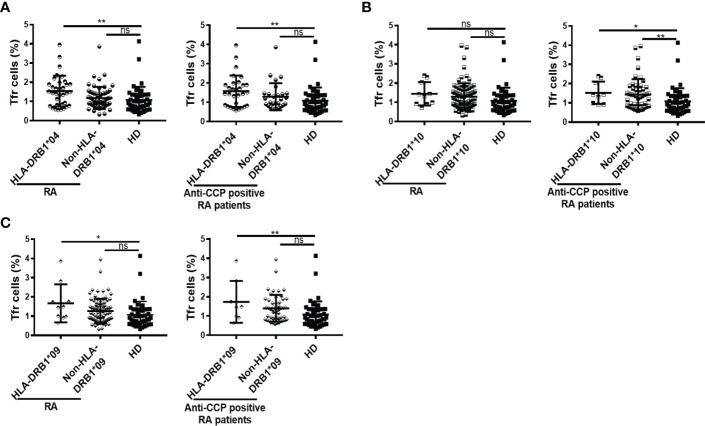
Frequency of Tfr cells according to HLA-DRB1 variants in RA patients. **(A)** Frequency of Tfr cells in RA patients (n=94) and in anti-CCP positive RA patients (n=58) divided by HLA-DRB1*04 presence (n=34 out of 94 RA patients and n=30 out of 58 patients with anti-CCP) and HD (n=43). **(B)** Frequency of Tfr cells in RA patients (n=94) and in anti-CCP positive RA patients (n=58) divided by HLA-DRB1*10 presence (n=10 out of 94 RA patients and n=9 out of 58 patients with anti-CCP) and HD (n=43). **(C)** Frequency of Tfr cells in RA patients (n=94) and in anti-CCP positive RA patients (n=58) divided by HLA-DRB1*09 presence (n=10 out of 94 RA patients and n=8 out of 58 patients with anti-CCP) and HD (n=43). Symbols represent individual subjects. Mean ± DS is shown. *P* values were determined by One-way Analysis of Variance (ANOVA) followed by Bonferroni Multiple Comparisons Test in **(A)**, **(B)** all RA patients and **(C)**. Kruskal-Wallis Test followed by Dunn’s Multiple Comparisons Test were used in **(B)** anti-CCP positive RA patients. Two-tailed probability *p < 0.05, **p < 0.01, ns, not significant.

We also evaluated possible associations between the frequency of Tfr cells and particular HLA-DRB1 variants significantly enriched in RA patients versus HD. Thus, we determined that patients with the HLA-DRB1*04 had a higher frequency of Tfr cells than HD, and this was found in the whole cohort as well as in patients with anti-CCP antibodies (**Figure 5A**). When the patients were divided according to the presence or absence of the HLA-DRB1*10, statistically significant differences were observed only in the patients that had anti-CCP autoantibodies. These patients presented a higher frequency of Tfr than HD, irrespective of the presence of HLA-DRB1*10 (**Figure 5B**). The frequency of Tfr cells was also significantly increased in patients with HLA-DRB1*09 compared to HD, not only in the whole cohort of RA patients but also in the group of patients with anti-CCP antibodies (**Figure 5C**).

### Relationships between tTfh and Tfr cells and the activity of the disease

**Figure 6:**
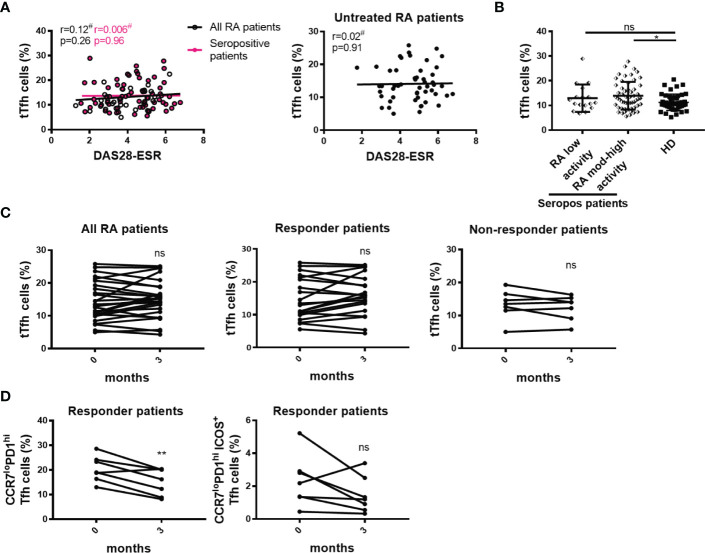
Relationship between tTfh cells and the activity of the disease. **(A)** Correlation between DAS28-ESR and tTfh cell frequency in all RA patients (n=99, empty black circles and line) or in only seropositive patients (n=72, pink circles and line) (left), and in untreated RA patients (n=52) (right). **(B)** Frequency of tTfh cells in seropositive RA patients (Seropos, n=72) with low (n=17) or moderate-high (n=55) disease activity and HD (n=46). **(C)** Frequency of tTfh cells at baseline (0 months) and after 3 months of treatment in all RA patients (n=27) (left), in responder (n=20, middle) and non-responder (n=7, right) RA patients. **(D)** Frequency of CCR7^lo^PD1^hi^ Tfh cell subset (left) and activated CCR7^lo^PD1^hi^ICOS^+^ Tfh cell subset (right) at baseline (0 months) and after 3 months of treatment in responder RA patients (n=7). Symbols represent individual subjects. Mean ± DS is shown in **(B)**. ^#^Pearson’s correlation coefficient r and *P* values are shown in **(A)**. Lines of best fit were drawn. One-way Analysis of Variance (ANOVA) followed by Bonferroni Multiple Comparisons Test were calculated in **(B)**. *P* values were determined by Paired samples t-test in **(C)** and **(D)**. Two-tailed probability *p < 0.05, **p < 0.01, ns, not significant.

Considering that RA patients exhibit different inflammatory status and disease activity, we next decided to examine a possible association between the frequency of Tfh cell subsets and DAS28-ESR. We found that the frequencies of tTfh cell and Tfh cell subsets showed no correlation with DAS28-ESR when RA patients were analyzed as a whole or selected according to seropositivity (**Figure 6A**; **S5A**). There was also no correlation between the frequency of tTfh cells and DAS28-ESR in untreated RA patients (**Figure 6A**). When the seropositive RA patients were divided according to the grade of disease activity, we observed that patients with moderate/high disease activity, but not those with low disease activity, exhibited a significantly higher frequency of tTfh cells than HD (**Figure 6B**). In contrast, seropositive RA patients with low and moderate/high disease activity showed a similar frequency of Tfh cell subsets to HD (**Figure S5B**).

To establish whether changes in Tfh cell frequency were associated with treatment efficacy, we evaluated the frequency of tTfh cells at baseline and 3 months after therapy. Analysis was performed in the whole cohort as well as in RA patients that were classified as responders and non-responders after the follow-up evaluation of the clinical response, as indicated in Materials and Methods. No significant differences in the frequency of tTfh cells were detected after treatment in the different groups of patients analyzed (**Figure 6C**). In a small cohort of 7 patients with good/moderate response, we extended this longitudinal evaluation to Tfh cell subsets and observed that the frequency of the CCR7^lo^PD1^hi^ Tfh cells was significantly reduced (**Figure 6D**) while the frequency of the activated CCR7^lo^PD1^hi^ICOS^+^ Tfh cell subset tended (p=0.05) to decrease after treatment without reaching statistical significance (**Figure 6D**).

**Figure 7:**
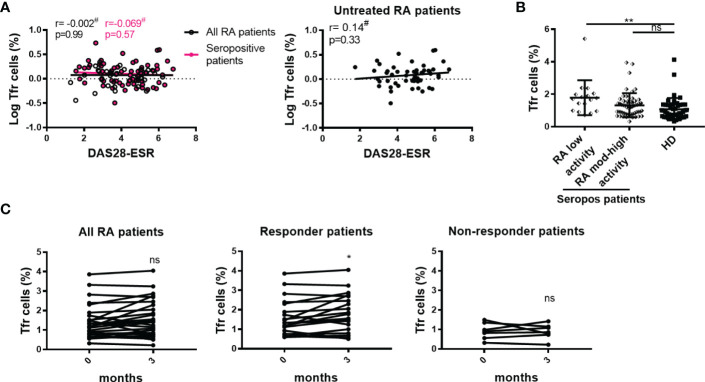
Relationship between Tfr cells and the activity of the disease. **(A)** Correlation between DAS28-ESR and Tfr cell frequency in all RA patients (n=99, empty black circles and line) or in seropositive patients (n=72, pink circles and line left), and in untreated RA patients (n=52, right). **(B)** Frequency of Tfr cells in seropositive RA patients (Seropos, n=72) with low (n=17) or moderate-high (n=55) disease activity and HD (n=46). **(C)** Frequency of Tfr cells at baseline (0 months) and after 3 months of treatment in all RA patients (n=27) (left), in responder (n=20) (middle) and non-responder (n=7) (right) RA patients. Symbols represent individual subjects. ^#^Pearson’s correlation coefficient r and *P* values are shown in **(A)**. Lines of best fit were drawn. Mean ± DS is shown in **(B)**. One-way Analysis of Variance (ANOVA) followed by Bonferroni Multiple Comparisons Test were calculated in **(B)**. *P* values were determined by Paired samples T-Test in **(C)**. Two-tailed probability *p < 0.05, **p < 0.01, ns, not significant.

Similarly to our findings with the frequency of tTfh cells and disease activity, we observed a lack of significant correlations between the frequency of Tfr cells and DAS28-ESR when analyzing the complete cohort, seropositive patients, or untreated patients (**Figure 7A**). We noted that seropositive patients with low disease activity presented increased Tfr cells frequency when compared to HD (**Figure 7B**). Longitudinal analysis showed that patients with a good/moderate response after 3 months therapy increased the frequency of Tfr cells, while non-responder patients presented unchanged Tfr cell frequencies (**Figure 7C**).

Additional studies with a larger number of samples should be carried out to confirm this result.

## Discussion

In agreement with a wealth of data showing an expansion of circulating Tfh cells in RA patients (19, 20, 22, 29, 38–46), we report here an increased frequency of tTfh cells, defined as CD25^lo^CD127^hi^CXCR5^+^CD45RA^neg^ CD4^+^CD3^+^ cells, in blood from RA patients, particularly those that present autoantibodies and are untreated. In addition, we detected a high frequency of CCR7^lo^PD1^hi^ and IL-17^+^IFNγ^neg^ Tfh cell subsets in seropositive RA patients. The Tfh cells with these phenotypes were reported to be efficient B cell helpers and were found altered in subjects with RA and other autoimmune diseases (10, 12, 44).

Of note, the notion that Tfh responses are increased during RA and other autoimmune diseases is predominant in the field (47, 48), although contrasting results have also been reported. Thus, Penatti et al. (49) observed lower frequencies of ICOS^+^cTfh cells in patients with RA, while other groups reported a conserved frequency of circulating Tfh cells in RA patients (24, 50). These differences are likely due to the heterogeneity of patient cohorts in terms of demographics, treatment, and disease activity, as well as to the non-homogeneous phenotypic definition of Tfh subsets. Remarkably, previous data from our laboratory showed no differences between RA patients and HD in the percentages of circulating Tfh cells or Tfh1, Tfh2, and Tfh17 subsets (23). Although the preceding and the current study were probably performed with patients with similar features, the differences in the results could be a consequence of the lower number of patients evaluated previously as well as of the more precise phenotypic identification of Tfh cells in the present work. These observations together with other reports in the same direction underscore the importance of establishing a consensus about Tfh cell characterization in terms of phenotype and nomenclature to allow the cross-comparison of works performed by different groups (16).

As a relevant counterpart of Tfh cells regarding B cell help, we also evaluated the frequency of Tfr cells in our cohort and found increased frequency of this subset in the blood of RA patients, particularly in seropositive untreated individuals. These results are in agreement with one study that evaluated a small cohort of patients in China (19) and the report of high levels of circulating Tfr cells in stable remission RA patients compared with active RA patients and controls (29). In contrast, data from Pandya et al. (50) using multivariate analysis indicated conserved Tfr frequencies in untreated patients with early RA, while other works reported decreased frequencies of circulating Tfr cells in RA patients regardless of disease activity (38, 39). As discussed before for Tfh cells, these conflicting results are likely consequences of different strategies used to characterize Tfr cell populations, together with the particularities of each cohort regarding clinical presentation and disease. In this regard, accumulating studies with clearly defined patient cohorts and an identification of follicular T cell subsets according to a consensus phenotype may provide the evidence required to more clearly understand the dynamics of these subsets in RA.

Interestingly, we detected a weak positive correlation between the frequency of Tfr and Tfh cells in seropositive RA patients, suggesting that increases in Tfr and Tfh cell frequencies are associated. Thus, the Tfr/Tfh ratio remains unaltered in total, seropositive, and seronegative RA patients in comparison to HD. These results support the hypothesis that the increase in Tfh cell responses may trigger an accumulation of Tfr cells, in an attempt to regulate exacerbated Tfh function. Interestingly, Chen Liu et al. (29) found an increased Tfr/Tfh ratio in stable remission RA patients compared to active RA and controls. Also, a decreased Tfr/Tfh ratio (or increased Tfh/Tfr ratio) has been observed in RA patients (19, 39). Altogether, the available data highlight alterations in the dynamics of Tfh and Tfr cells and a disruption in their balance, which may be involved in the pathogenesis or progression of RA as will be discussed later.

To understand the dynamics of Tfh and Tfr cells at the level of an effector RA site, we analyzed the frequencies of these subsets in SF. We detected a lower frequency of tTfh cells in SF versus the paired PB sample from the same patient. Other reports depict a similar scenario (45), as well as a contrasting result showing increased PD1^hi^Tfh cells frequency in SF in comparison to PB of RA patients (21). Besides the already discussed factors that may lead to conflicting data about follicular T cell subsets, an additional aspect that may explain variable Tfh cell presence in SF is the presence of ectopic lymphoid structures (ELS) in the synovial membrane. ELS that sustain autoreactive B cell activation and autoantibody production locally (51) have been shown to contain Tfh cells and, in particular, both CD4^+^PD-1^hi^CXCR5^+^ Tfh cells and peripheral CD4^+^PD-1^hi^CXCR5^neg^ T cells were found adjacent to B cells (25). So, it could be hypothesized that the scarce number of Tfh cells detected in SF from our patients is a consequence of their migration to the ELS. Thus, the length of disease evolution of each patient may affect the kinetics of cell migration within the joint and therefore account for the variability of Tfh cell frequencies reported in the different works. Remarkably, while the frequency of Tfh cells in SF was reduced with respect to PB, Tfr cell abundance was similar in both biological fluids, indicating a possible enrichment in follicular regulatory functions within the synovia aimed at counteracting processes occurring within the ELS as suggested (52).

Many reports evaluating follicular T cell subsets in RA found a positive correlation between the frequency of circulating Tfh cells and the titers of autoantibodies, including anti-CCP Abs and/or RF (20, 22, 38, 42, 43, 53). Moreover, many works found a positive correlation between Tfh cell subsets and disease activity measured by DAS28 (20, 41, 45, 53). In contrast, such correlations were not observed in our cohort of patients nor in the recent report from Su et al. (46), likely due to the fact that untreated patients had a high disease activity and that the levels of autoantibodies did not correlate with DAS28 (54). However, it is important to underline that the fact that seropositive but not seronegative patients from our cohort present significantly higher frequencies of total and CCR7^lo^PD1^hi^ Tfh cells in comparison to HD suggests that the imbalance in Tfh subsets, although not directly and linear proportionally related to autoantibody titers, may potentiate the autoantibody response. In addition, our data also suggest that such a Tfh cell imbalance may be involved in the pathogenesis of RA, as patients with moderate/high disease activity exhibited a higher frequency of tTfh cells.

When analyzing the regulatory follicular compartment, we found a weak positive correlation between Tfr cell frequency and the levels of anti-CCP antibodies. Although contradictory at first glance, Fonseca et al. (28) also found augmented blood Tfr cell frequency in primary sjögren´s syndrome patients, another autoimmune disease characterized by autoantibody production. These authors described that human circulating Tfr cells are not fully licensed with B cell-suppressive function as are their lymphoid tissue counterparts, and therefore Tfr cell increases in blood are associated with ongoing humoral responses rather than with the suppression of Ab production. Nonetheless, other authors found that the frequency and numbers of Tfr cells in RA patients were negatively correlated with the levels of IgG, RF and anti-CCP antibodies, suggesting that Tfr cells can suppress humoral autoimmunity in RA patients (29, 38). In addition, the frequency of Tfr cells was found to negatively correlate with DAS28 in several reports (29, 38, 39). In this direction, our data show an increased frequency of Tfr cells in seropositive RA patients with low disease activity, supporting the idea that Tfr cells may be involved in the control of disease progression. Beyond the prevailing model for Tfr cell function repressing excessive Tfh and GC B cell proliferation, the full range of Tfr cell roles may include helper functions (55). Given the conflicting data about the correlation between the titer of autoantibodies and RA disease activity, the exact mechanisms by which Tfr cells may promote disease control during RA remains obscure and deserves further evaluation.

Among the major genetic susceptibility loci for RA, HLA-DRB1 that contain the shared epitope have been associated with the development of ACPA and bone destruction (2, 8, 56). Of note, although it is assumed that HLA-DRB1 molecules may contribute to the development of antibody responses by presenting antigenic peptides to helper T lymphocytes, their association with T cell subsets remains unknown. Here, we reported that the presence of HLA-DRB1*04 and *10 is not related to changes in the frequency of Tfh cells, but that HLA-DRB1*09 is associated with a higher frequency of Tfh cells in RA patients. In contrast, the increased frequency of Tfr cells was associated with HLA-DRB1*09 as well as HLA-DRB1*04 presence, not only in the whole cohort of RA patients but also in the group of patients with anti-CCP antibodies. To our knowledge, this is the first report of associations between HLA-DRB1 variants and T cell subsets able to provide B cell help. Although the implications of these associations remain to be understood, a previous antecedent by Nagafuchi et al. (53) described that RA patients with one or more HLA-DRB1 locus with shared epitope displayed a significantly higher frequency of activated memory CXCR4^+^CD4^+^ T cells, correlating with higher disease activity. In our work, we were not able to establish such associations, but our results provide valuable incremental data for elucidating the molecular contribution of HLA genotypes in cellular and humoral responses that participate in the pathogenesis of RA.

Finally, the potential use of Tfh and Tfr cells as biomarkers during RA prompted us to evaluate the impact of RA treatments in these populations. Our longitudinal analysis after 3 months of treatment showed no changes in tTfh cell frequencies regardless of treatment success, in agreement with previous reports that showed no decrease in Tfh cell levels in patients undergoing 24 weeks of DMARD therapy (41), and unchanged circulating Tfh numbers/percentage in RA patients experiencing significant clinical improvement at 12 months of oral MTX therapy (44). However, we identified changes in response to treatments when analysis was focused in particular Tfh cell subsets. Thus, we observed a decreased frequency of CCR7^lo^PD1^hi^ cell subsets at 3 months of treatment in a small cohort of patients who successfully responded to therapy. A similar behavior was observed with CCR7^lo^PD-1^hi^ICOS^+^Tfh cells (p=0.05). Similarly, a significant reduction was observed in the percentages of ICOS^+^ Tfh cells following 1 month of DMARDs and *T. wilfordii* (Chinese herb) treatments in drug-responding patients (20). Effector memory Tfh cells and their subfractions decreased over time in patients who started treatment with MTX or biologics (45). Interestingly, we also observed an increased frequency of Tfr cells in responders to treatment. Therefore, we hypothesize that the Tfr cell increase may be responsible for the reduction in frequency of the Tfh cell subsets after 3 months of treatment in patients showing successful therapy response. This, together with the fact that Tfr cells were increased in seropositive RA patients with low disease activity, suggests that targeting regulatory factors and pathways involved in Tfr differentiation and/or function may favor disease remission. Remarkably, we observed that patients under prolonged treatment with TNF inhibitor exhibited increased frequency of Tfr cells, highlighting a possible role of the TNF pathway in Tfr cell development, as suggested by Santinon et al. (57) which showed that anti-TNF treatments increase a particular subset of Tregs expressing TNF-RII in RA patients.

Altogether, our results provide novel as well as accumulative data that link the HLA-DRB1 genotype, the frequencies and phenotypic characterization of Tfh cells and Tfh cell subsets, autoreactive humoral responses, disease activity, and response to treatment in patients with RA.

Although our data show certain limitations as a result of the small number of patients who did not respond to treatment, the information obtained is a valuable addition for completing the emerging picture of the role of effector and regulatory cellular responses specialized in B cell help in the context of autoimmune diseases.

## Data availability statement

The original contributions presented in the study are included in the article/**Supplementary Material**. Further inquiries can be directed to the corresponding authors.

## Ethics statement

The studies involving human participants were reviewed and approved by HNC Institutional Committee of Ethics of Health Research and the Council for Ethical Evaluation of Health Research of the Cordoba government. The patients/participants provided their written informed consent to participate in this study.

## Author contributions

PF performed and designed most of the experiments, analyzed data and prepared the figures and manuscript. LIO, CA, EZ, NP, and AC collaborated with performing experiments. TA and LM supervised the design, analysis, and interpretation of the HLA typing. EM, LBO, IC, and MW contributed to the recruitment and clinical evaluation of the patients. CM contributed to the study analysis and result discussion. PF, AG, and EA conceived, designed, and supervised the study and wrote the manuscript. All authors reviewed the manuscript before submission.
