# Supplementary figures and images for: Dynamics of circulating follicular helper T cell subsets and follicular regulatory T cells in rheumatoid arthritis patients according to HLA-DRB1 locus
Source: Front Immunol. 2022 Dec 13;13:1000982. doi: 10.3389/fimmu.2022.1000982 (PMC9793086; doi:10.3389/fimmu.2022.1000982)

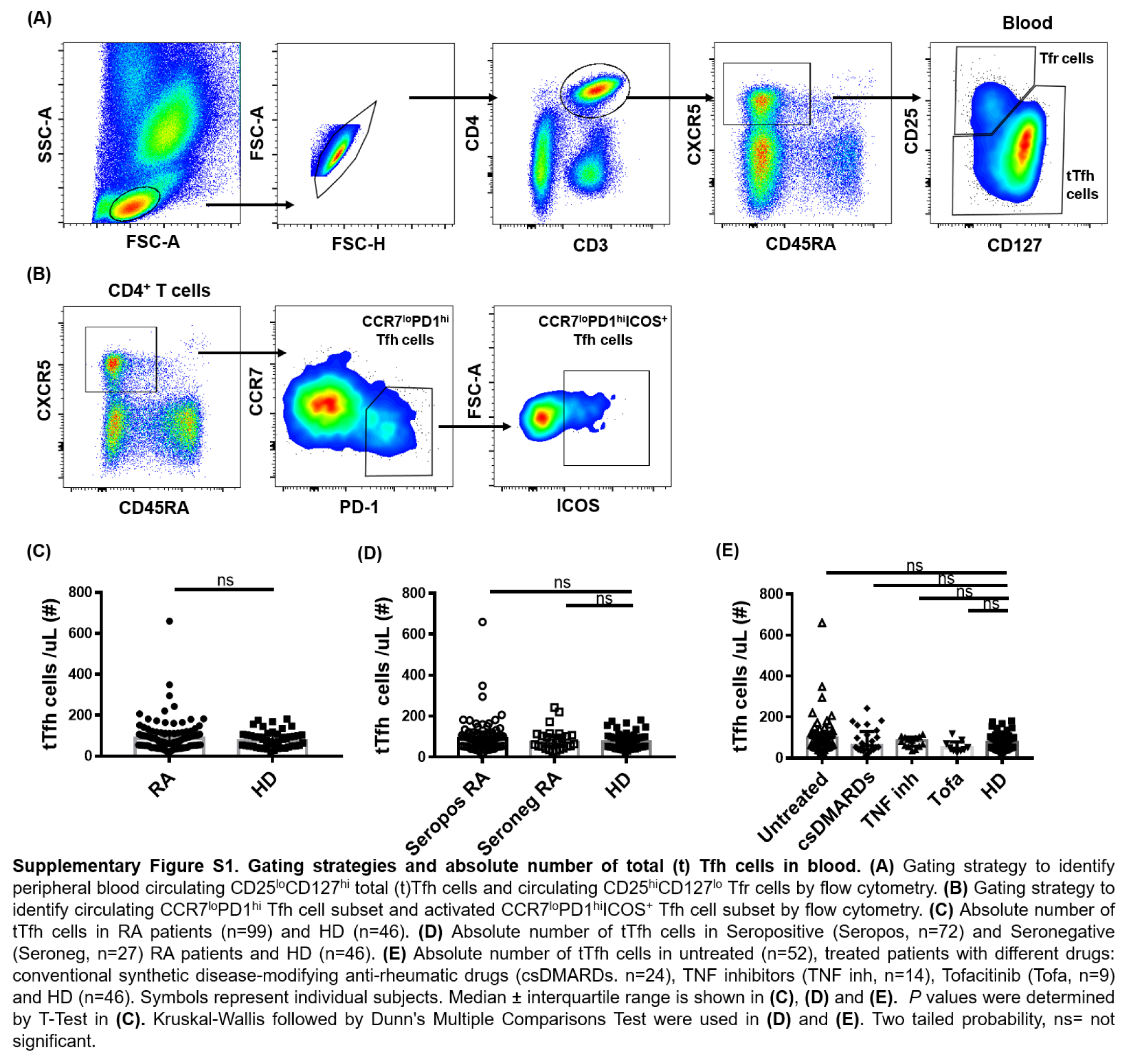

Supplement: Supplementary file 1 [file Image_1.tif]

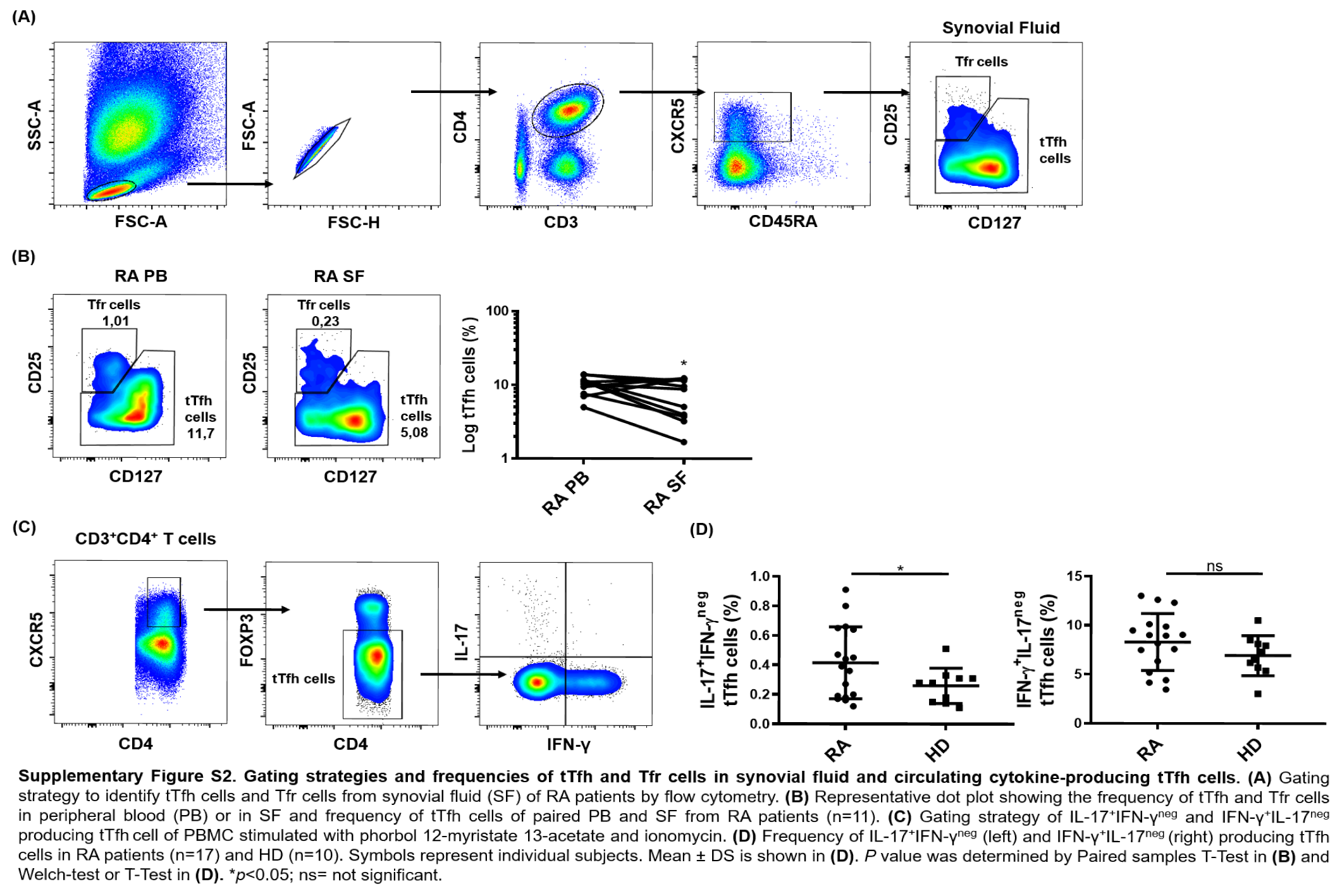

Supplement: Supplementary file 2 [file Image_2.tif]

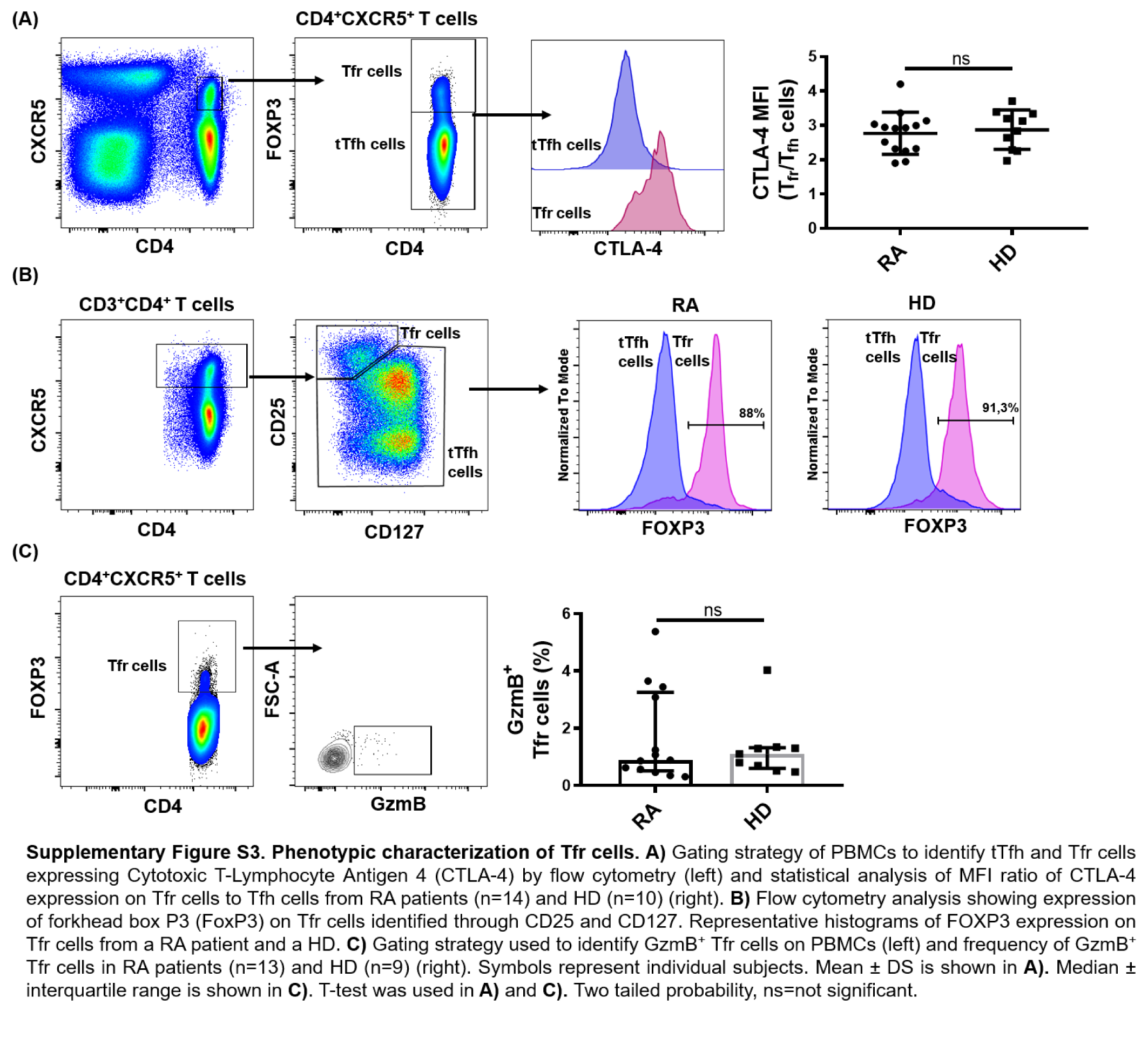

Supplement: Supplementary file 3 [file Image_3.tif]

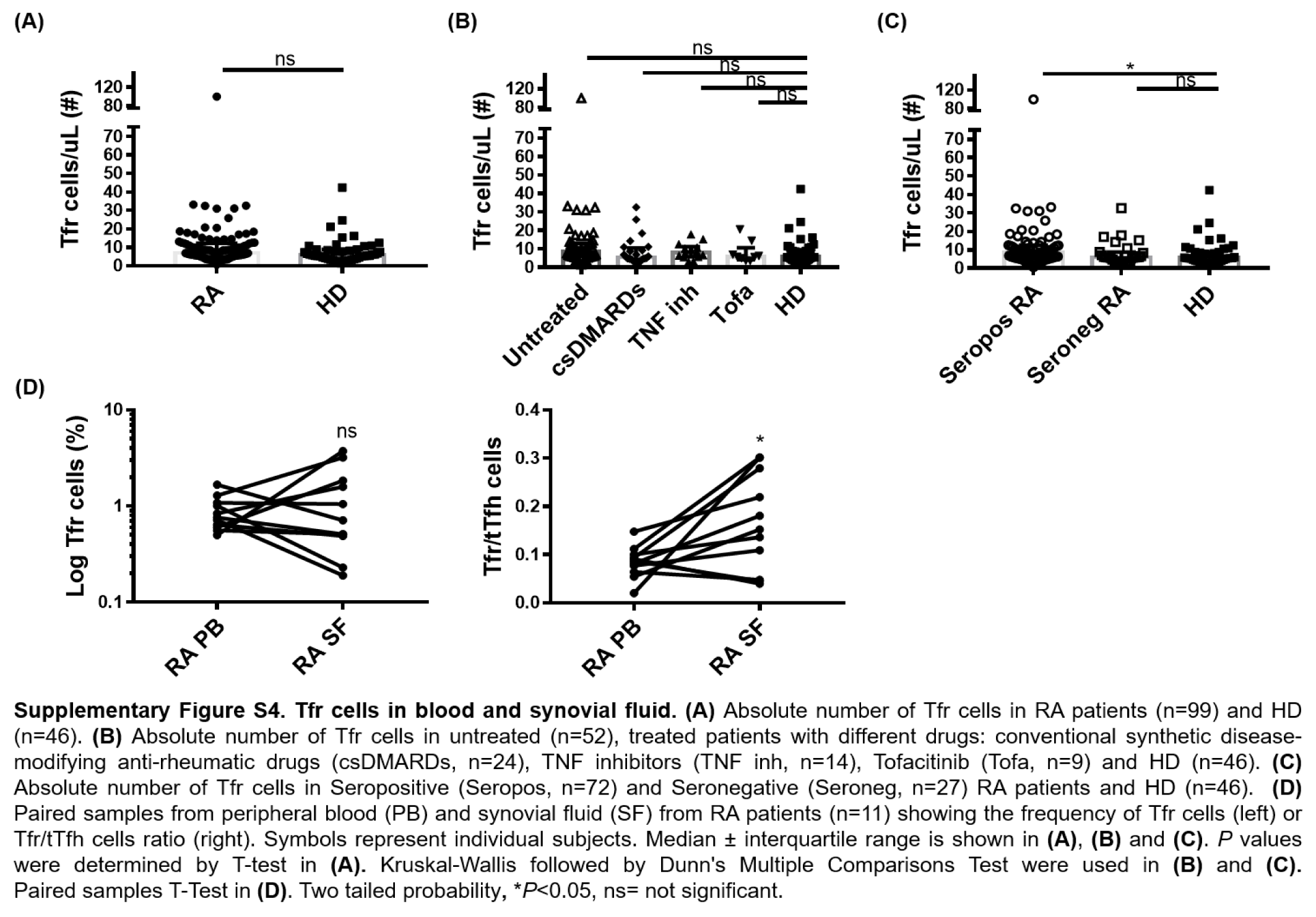

Supplement: Supplementary file 4 [file Image_4.tif]

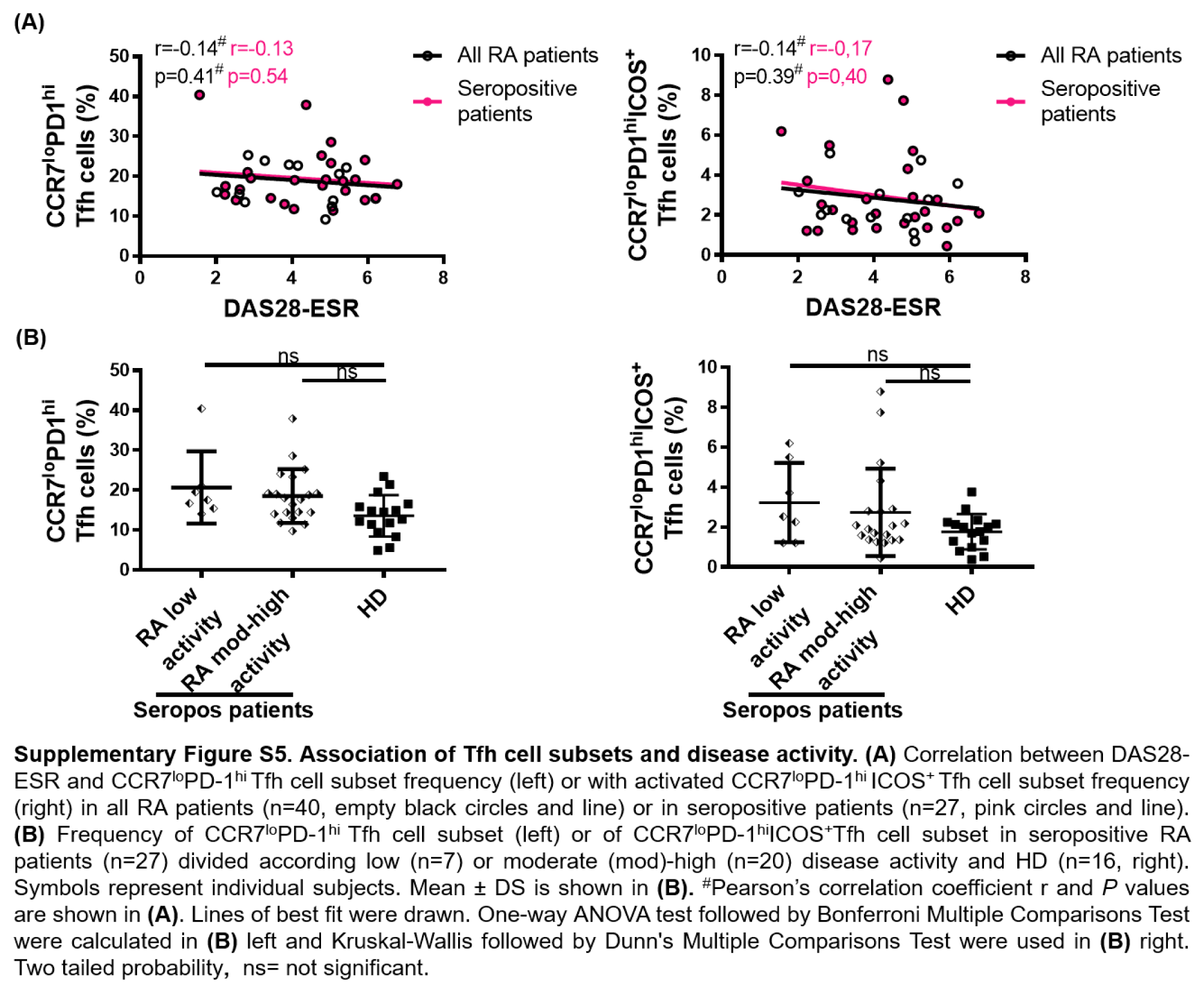

Supplement: Supplementary file 5 [file Image_5.tif]
