# Supplementary material for: Dynamics of circulating follicular helper T cell subsets and follicular regulatory T cells in rheumatoid arthritis patients according to HLA-DRB1 locus
Source: Front Immunol. 2022 Dec 13;13:1000982. doi: 10.3389/fimmu.2022.1000982 (PMC9793086; doi:10.3389/fimmu.2022.1000982)
Supplement: Supplementary file 6 [file Table_1.docx]

| **Supplementary Table 1. Antibodies and immunostaining panels used for flow cytometry** | | | | |
| --- | --- | --- | --- | --- |
| **Immunostaining panel** | **Antibody** | **Fluorochrome** | **Clone** | **Manufacturer** |
| 1. **Tfh and Tfr cell immuno-phenotyping (whole blood and synovial fluid)** | CD3 | FITC | UCHT1 | BD Pharmigen*^a^* |
|  | CD4 | APC-H7 | RPA-T4 | BD Pharmigen |
|  | CD45RA | PE-Cy7 | 5H9 | BD Pharmigen |
|  | CXCR5 (CD185) | AF647 | RF8B2 | BD Pharmigen |
|  | CD25 | PE | M-A251 | BD Pharmigen |
|  | CD127 | PerCpCy5.5 | eBioRDR5 | Invitrogen*^b^* |
| 1. **Tfh cell subsets immuno-phenotyping (whole blood)** | CD4 | APC-H7 | RPA-T4 | BD Pharmigen |
|  | CD45RA | FITC | ALB11 | BeckmanCoulter^c^ |
|  | CXCR5 (CD185) | AF647 | RF8B2 | BD Pharmigen |
|  | CCR7 (CD197) | PE-Cy7 | 3D12 | BD Pharmigen |
|  | PD-1 (CD279) | PE | eBioJ105 | Invitrogen |
|  | ICOS (CD278) | PerCp eF710 | ISA-3 | Invitrogen |
| 1. **IFNγ/IL-17 (stimulated PBMCs)** | CD3 | FITC | UCHT1 | BD Pharmigen |
|  | CD4 | APC-H7 | RPA-T4 | BD Pharmigen |
|  | CXCR5 (CD185) | AF647 | RF8B2 | BD Pharmigen |
|  | FOXP3 | PE-Cy7 | 236A/E7 | Invitrogen |
|  | IFN-γ | PerCpCy5.5 | B27 | BD Pharmigen |
|  | IL-17A | PE | N49-653 | BD Pharmigen |
| 1. **CTLA-4/GzmB (Unstimulated PBMCs)** | CD4 | APC-H7 | RPA-T4 | BD Pharmigen |
|  | CXCR5 (CD185) | AF647 | RF8B2 | BD Pharmigen |
|  | FOXP3 | PE-Cy7 | 236A/E7 | Invitrogen |
|  | CTLA-4 (CD152) | PE | L3D10 | Biolegend*^d^* |
|  | GzmB | FITC | GB11 | Biolegend |
| *^a^* BD Pharmingen^TM^, San José, United States. *^b^*  Invitrogen, Carlsbad, San Diego, United States. *^c^* Beckman Coulter Company, Marseille, France. *^d^* Biolegend, San Diego, United States. PBMCs: peripheral bood mononuclear cells; FITC, fluorescein isothiocyanate; AF, Alexa Fluor; APC, allophycocyanin; Cy, cyanine; PE, phycoerythrin; PerCP, peridinin-chlorophyll proteins | | | | |
