# Supplementary material for: Dynamics of circulating follicular helper T cell subsets and follicular regulatory T cells in rheumatoid arthritis patients according to HLA-DRB1 locus
Source: Front Immunol. 2022 Dec 13;13:1000982. doi: 10.3389/fimmu.2022.1000982 (PMC9793086; doi:10.3389/fimmu.2022.1000982)
Supplement: Supplementary file 7 [file Table_2.docx]

| **Supplementary table 2. Correlation between Rheumatoid Factor titers and different T follicular cell populations** | | | | |
| --- | --- | --- | --- | --- |
|  | **RF positive RA patients** | | **RF positive untreated RA patients** | |
|  | r | *p* value | r | *p* value |
| **tTfh cells (%)** | 0.025*^a^* | 0.84 | 0.069*^a^* | 0.67 |
| **CCR7^lo^PD1^hi^ Tfh cell subset (%)** | 0.087*^a^* | 0.66 | 0.10*^a^* | 0.69 |
| **CCR7^lo^PD1^hi^ICOS^+^Tfh**  **cell subset (%)** | 0.011*^a^* | 0.96 | 0.37*^a^* | 0.14 |
| **Tfr cells (%)** | 0,081*^a^* | 0,51 | 0,18*^a^* | 0,27 |
| ***^a^***Spearman's coefficient of rank correlation (rho).  RA= Rheumatoid Arthritis; RF=Rheumatoid Factor (RV<1/20) | | | | |
